# Risky sexual behaviour amidst predicament of acceptable sexually transmitted infection partner notification modalities: A cross-sectional survey amongst minibus taxi drivers in Gauteng Province, South Africa

**DOI:** 10.4102/safp.v62i1.5165

**Published:** 2020-12-10

**Authors:** Mathildah M. Mokgatle, Sphiwe Madiba

**Affiliations:** 1 Department of Biostatistics, School of Public Health, Sefako Makgatho Health Sciences University, Pretoria, South Africa; 2 Department of Environmental and Occupational Health, School of Public Health, Sefako Makgatho Health Sciences University, Pretoria, South Africa

**Keywords:** partner notification, sexually transmitted infections, STI prevention and control, taxi industry, risky sexual behaviour, short message service

## Abstract

**Background:**

Sexually transmitted infection patient-initiated partner notification (PN) approach has been implemented over a decade in South Africa, however, use and update by patients has been limited. This study assessed the perceived use of patient-initiated PN by using referral slips and measured the level of acceptability of provider-initiated PN by using short message service (SMS) to the personal mobile phones of sexual partners.

**Methods:**

A formative evaluation approach using a quantitative survey amongst 722 minibus taxi drivers in nine major taxi ranks in Tshwane Municipality, Gauteng Province. STATA IC version 13 was used for data analyses.

**Results:**

The mean age of the participants was 37.2 years (59%) were single, 59.5% had multiple sexual partners, 52.2% did not use a condom during the last sexual act, 42.8% reported inconsistent use of condoms and 65% tested for HIV in the past 12 months. The majority (98.2%) understood the importance of PN, but 51% would prefer telling a sexual partner face to face. Perceived easiness of delivering a PN slip was 69.1%, and 93% would use a PN slip received from a partner. Acceptability of provider-initiated PN by using an SMS was 62.7% and about a third (32.5%) were not in favour of provider-initiated PN by SMS. Twenty four point 7 percent (24.7%) preferred patient-initiated PN and 24.3% preferred provider-initiated PN.

**Conclusion:**

Preferred patient-initiated and provider-initiated PN was almost equal, hence, the provider-initiated PN should be augmented to support the current patient-initiated PN to increase the overall STI notification.

## Introduction

South Africa’s burden of disease because of sexually transmitted infections (STIs) is currently one of the largest in the world, and this is true for all STIs, including human immunodeficiency virus (HIV) and human papilloma virus.^1^ In the African region in 2016, amongst men aged 15–49 years, the estimated prevalence of chlamydia was 4.0% (2.4% – 6.1%), gonorrhoea was 1.6% (0.9% – 2.6%), syphilis was 1.6% (1.2% – 2.0%) and trichomonas was 1.2% (0.7% – 1.8%).^2^ The significantly high prevalence of STIs in sub-Saharan Africa (SSA) poses a threat because of the increased risk of HIV transmission.^3^ From 2010 to 2011, the STI incidence for South Africa was 3.9%,^4^ and the prevalence of syphilis and male urethral discharge was 8.3% and 13.8%, respectively.^5^

Key drivers of STIs include risky sexual behaviour, such as multiple sexual partners, transactional sex, vulnerability of women in sexual relationships and the high rate of concurrent partnerships.^6,7,8,9^ Risky sexual behaviour and the asymptomatic nature of STIs during the early stages of the infection make the control and treatment of STIs complicated.^8^ The silent nature of STIs presents a huge public health threat; hence, STI partner notification (PN) and referral are an important public health approach to reduce the burden.^8^ Partner notification and referral are beneficial in controlling the spread of STIs if done correctly; however, occasional lack and/or shortage of PN and referral slips in the health facilities further compromises STI control.^10,11^ Moreover, the use of electronic communication like short message service (SMS) can facilitate PN^9^ given the increased number of people globally who have access to mobile phones.^12^

Even though the PN approach has been implemented for more than a decade, since the implementation of the syndromic management of STIs protocol,^13^ it has limitations that affect the intention to control the spread of STIs. The approach is limited because of under-reporting of the number of sexual partners by the patients, which results in healthcare workers (HCWs) issuing fewer notifications and referral slips.^14,15^ Under-reporting occurs because of the reluctance to openly discuss sexual issues, the biological nature and characteristics of the STIs and the fear of moral judgement.^1,16^ Failure to inform sexual partners of their exposure to STIs increases the risk of transmission to other sexual partners who remain asymptomatic, and continuous infection of new partners and re-infections.^17^

The effective treatment and control of STIs depend on screening to detect and treat STIs amongst the sexual partners of the patients with STI. This is further dependent on the patient-initiated PN practices by using referral slips. A systematic review revealed that face-to-face patient-initiated PN interventions have had limited success, which reflects discordance between high levels of acceptability of PN and low rates of utilization.^10^ This is despite a high level (70.2%) of PN counselling by HCWs and high acceptability and possible recommendation of PN to sexual partners.^18^ In developing countries, PN and referral practices by using referral slips fail to reach the majority of partners.^8,19,20^

In addition to the patient-initiated PN practices, provider-initiated PN such as text messaging, the Internet and phone calls are promising strategies to expand PN services.^10,11,12^ The benefits of provider-initiated PN are that electronic messages may enhance rapid notification because they can reach partners who may be geographically dispersed, are likely to be used with partners who may not be notified otherwise and come at a low cost.^12^ Provider-initiated PN by using SMS presents an additional and promising strategy for the control and prevention of STIs in South Africa.^11,14,15^

Minibus taxi drivers were selected because the focus of existing research has been on the assessment of HIV infection in the transport industry amongst truck drivers in Gauteng Province and in South Africa generally.^21^ Research indicates that men in the transport industry have high sexual risk behaviours and low HIV risk perceptions.^21,22^ Furthermore, minibus taxi drivers have poor access to health screening facilities because of the nature of their work, which may have an impact on STI treatment and PN.^23^ Being highly mobile or travelling long distances impact on the ability to be able to reach the sexual partner for referral for STI treatment.^21^

This article presents an assessment of perceived use of patient-initiated PN by using a referral slip from the sexual partner and the acceptability of provider-initiated PN by using SMS to the personal mobile phones of the sexual partners. The survey focus was on self-reported sexual behaviours, knowledge of STI symptoms and acceptability of provider-initiated PN amongst male minibus taxi drivers who have access to the syndromic management of STIs.

## Methods

### Design

A quantitative cross-sectional survey by using anonymous structured researcher-assisted questionnaires was conducted.

### Study setting and population

The taxi industry employs more than 600 000 people and transports 15 million commuters per day, which is an equivalent of 65% of public transport commuters. A typical layout of the industry consists of a series of taxi ranks, hubs of the greater system, where hundreds of taxis line up to transport people to all parts of the city and beyond.^24,25,26^ Tshwane District in Gauteng Province is one of the large cities that has a complex network of taxi ranks transporting passengers to and from the city, urban areas and peri-urban areas. The majority of the taxi ranks are situated near shopping malls, industrial areas, hospitals and business centres for ease of access by passengers. In Tshwane District, only a few taxi ranks are built as formal structures to enable exchange or transfers and articulation with other modes of transport such as busses or trains. Minibus taxis from smaller taxi ranks in the townships, suburbs and informal settlements merge to feed central taxi ranks, and passengers select their trips by choosing a relevant taxi to their destination.^26^

### Study population and sample

The survey was conducted in nine major minibus taxi ranks, and all drivers working in the selected ranks formed the study population. Systematic random sampling of taxis that were waiting to load passengers was performed. The first minibus taxi was randomly selected from a list of taxi queue controllers, the driver of the selected taxi became the first participant and then the driver of every third minibus taxi in the queue was requested to participate in the study. The population of drivers per taxi rank was approximately 110, and the total population was 990 drivers. A sample size calculated per taxi rank at 95% confidence level, 5% margin of error and 50% response distribution^26^ was a minimum of 86 taxi drivers per rank. The total sample size reached for nine taxi ranks was 722 taxi drivers.

### Data collection

Data collection was undertaken from March to July 2016 after the Taxi Association and other managers from the different taxi ranks granted permission. Data collection was scheduled to occur during off-peak hours so that the research process did not interrupt the work of the taxi drivers. Data were collected by a team of trained fieldworkers by using a researcher-administered structured questionnaire translated from English into local languages (Setswana, IsiZulu and Sepedi). The drivers were asked about whether they would use patient-initiated PN, the likelihood of telling a partner that they themselves were diagnosed with STI, whether they would deliver a PN notification and referral slip to their sexual partners, their acceptability of provider-initiated PN using an SMS and preference for notification if a partner was diagnosed with STI. The tool further captured socio-demographic data, sexual behaviour, condom use, multiple sexual partnerships and HIV testing history. The drivers were further asked a series of questions to assess the level of STI knowledge.

### Data analysis

Descriptive statistical analyses were conducted to describe demographics, sexual relationships, HIV testing practices, perceived use of referral slips from sexual partners and level of acceptability of provider-initiated PN by using SMS. Dependant variables were perceived use of referral slips from sexual partners and acceptability of provider-initiated PN by using SMS. The independent variables were demographic characteristics, sexual relationships, HIV testing history, condom use in the last sexual act and likelihood of telling a partner when diagnosed with STIs. All statistical analyses were performed by using STATAIC version 13.

## Results

A sample of 722 male minibus taxi drivers participated in the study, and they had been working in the minibus taxi industry for the mean period of 8.7 years (range, 1–40 years; standard deviation [SD] = 7.9 years). Their ages ranged from 19 to 68 years, with a mean age of 37.2 years (SD = 10.3 years). The majority (43.8%) had received some secondary education, but only 39.5% had completed the 12th grade and 8.6% had a tertiary education. Concerning living arrangements, 40.8% were married and only 36% lived with their wives in the same household whilst others left their wives and families and migrated for work in the City of Tshwane. On reports of sexual partners, 44.2% had two sexual partners and 15.3% had three or more sexual partners, more than half (52.2%) did not use a condom the last time they had sex and 17.5% reported never using a condom (Table 1).

**TABLE 1:** Demographics and sexual behaviour characteristics of minibus taxi drivers in Gauteng Province (*n* = 722).

Variables	Frequency	Percentage
**Age category** (missing = 10)		
19–24 years	43	6.0
25–34 years	284	40.0
35–44 years	232	32.0
45–54 years	93	13.1
> 54 years	59	8.3
**Age group** (missing = 10)		
Younger (19–34 years)	326	45.9
Older (35 years and older)	384	54.1
**Highest level of education** (missing = 1)		
No formal schooling	7	1.0
Primary school	51	7.1
Secondary school	316	43.8
Completed 12th grade	285	39.5
Tertiary education	62	8.6
**In a sexual relationship** (missing = 2)		
Yes	676	93.9
No	44	6.1
**Type of sexual relationship** (missing = 9)		
Married	291	40.8
Casual partners	325	45.6
Steady partners	63	8.8
**Number of sexual partners** (missing = 49)		
One partner	255	40.5
Two partners	278	44.2
Three or more partners	96	15.3
**Living arrangements** (missing = 6)		
Live with wife	260	36.3
Live with girlfriend	133	18.6
Live with family members	142	19.8
Living alone	181	25.3
**Used condoms at last sexual activity**		
Yes	337	47.8
No	368	52.2
**Overall condom use in the past 6 months** (missing = 22)		
Never use condoms	117	17.5
Always use condoms	265	39.7
Use condoms sometimes	286	42.8
**Ever tested for HIV**		
Yes	555	82.4
No	118	17.6
**Tested for HIV in the past 12 months** (missing = 12)		
Yes	459	65.0
No	246	35.0

HIV, human immunodeficiency virus.

### Awareness and knowledge of sexually transmitted infection symptoms

Knowledge of STIs symptoms is a predictor for early access of treatment and PN and referral. Almost all (96.5%) had awareness of STIs. For most (39.6%), the source of STI information was the clinic. Table 2 presents their responses about STI symptoms. Penile discharge as the most common STI symptom was reported by 44.6%. The level of knowledge of STI symptoms was low, as only 45.1% knew that men could have STIs without symptoms at an early stage of infection. The results also showed that self-reported STI occurrence was low, with only 5.5% reported to have been diagnosed with STIs in the past 12 months; this is despite the high occurrence of low usage of condoms amongst the study participants.

**TABLE 2:** Awareness and knowledge of sexually transmitted infection symptoms and source of sexually transmitted infection information.

Variable	Frequency	Percentage
**Ever heard of STIs**		
Yes	692	96.5
No	25	3.5
**Diagnosed with an STI in the past 12 months (missing values = 1)**		
Yes	40	5.6
No	680	94.4
**Common STIs in men**		
Itching in genital area	174	28.8
Discharge from penis	270	44.6
Pain when urinating	114	18.8
Genital ulcers	43	7.1
Pain during sex	4	0.7
**Men can have STIs without symptoms at an early stage of infection**		
Yes	242	34.2
No	319	45.1
Not sure	146	20.7
**Source of information about STIs**		
Clinic	276	39.6
Hospital	22	3.2
General practitioner	10	1.4
Media	178	25.5
Health campaigns	94	13.5
Word of mouth	117	16.8

STIs, sexually transmitted infections.

### Perceived use of patient-initiated partner notification of sexually transmitted infection

Table 3 shows the taxi drivers’ views about PN. Almost all (98.2%) reported that it was important to notify a sexual partner about an STI diagnosis. A large proportion of the participants (97.5%) were not sure whether they would verbally inform a sexual partner if they were diagnosed with STIs. In contrast, 93.2% would give a PN referral slip to a sexual partner if they were diagnosed with an STI, 69.5% felt that it would be easy to give a sexual partner an STI notification and referral slip and 91.5% said they would use a referral slip received from their sexual partner to access treatment.

**TABLE 3:** Perceived use of partner notification by using referral slip and acceptability of provider-initiated partner notification by using short message service.

Response	Frequency	Percentage
**If you were infected with STI, could you tell your partner about it?**		
Yes	18	2.5
No	698	97.5
**If you were diagnosed with STI, would you give your partner the STI PN slip?**		
Yes	670	93.2
No	40	5.6
Not sure	9	1.2
**Importance of telling a partner when infected with an STI**		
Not important	13	1.8
Important	317	43.9
Very important	392	54.3
**Perceived use of referral slip received from sexual partner (missing values = 4)**		
Yes	659	91.8
No	39	5.4
Not sure	20	2.8
**Perceived ease of delivering a referral slip to sexual partner**		
Easy	504	69.5
Not easy	194	26.9
Not sure	26	3.6
**Acceptability of SMS notification of STIs from HCWs (missing value = 1**)		
Yes	452	62.7
No	234	32.5
Not sure	35	4.8
**Reasons for non-acceptability of SMS notification of STIs** (*n* = 109)		
Prefer face-to-face disclosure	55	50.5
SMS is not reliable	47	43.1
SMS could cause conflict	7	6.4
**Preferred type of notification for STI referral (missing values = 1**)		
Patient-initiated using PN slip	175	24.3
Provide-initiated by SMS	178	24.7
Face to face by partner	368	51.0

HCW, healthcare workers; PN, partner notification; SMS, short message service; STI, sexually transmitted infection.

### Acceptability of provider-initiated partner notification of sexually transmitted infection

The level of acceptability of PN by using an SMS from a health care provider was 62.7%, but only 15% gave reasons why PN by using SMS was not acceptable and of those, 43% said SMS is not reliable and 6.4% said that SMS could cause conflict. These data show that the preferred approaches of PN amongst the taxi drivers are 51% telling the partner face to face, 24.7% SMS from clinic and 24.3% PN slip (Table 3).

## Discussion

The study assessed self-reported sexual behaviours, knowledge of STI symptoms and perceived use of patient-initiated PN and acceptability of provider-initiated PN amongst male minibus taxi drivers who have access to the syndromic management of STI. The mean age of minibus taxi drivers was 37.2 years and had been working in the taxi industry for the mean period of 8 years. Almost all of the minibus taxi drivers had awareness of STIs and accurately cited the STI symptoms. However, more than half did not know that men could have STIs without showing symptoms during the early stages of the infection. The critical point of not knowing that STIs are asymptomatic during the early stages of the infection explains the issue of missed opportunity in early infection, high STI transmission to sexual partners and high prevalence of STIs in the country. Not knowing the asymptomatic nature of STIs has implications for the health education and health promotion programmes to create awareness and accurate information on STI prevention, transmission, treatment and control.

Almost 20% of the minibus taxi drivers reported to never have used condoms during sexual intercourse, over half did not use a condom during the last sexual act and 42.8% reported inconsistent use of condoms. Moreover, two-thirds had more than one sexual partner and almost half (45.6%) were in casual sexual relationships. Other studies cited that inaccurate information and beliefs about STIs, having multiple sexual partners and risky sexual behaviour were common in male patients with STI as compared with female patients with STI.^14,18,20^

A high proportion of the minibus taxi drivers knew the importance of notifying a sexual partner once diagnosed with an STI. However, there were conflicting views regarding informing sexual partners face to face and delivering a PN and referral slip should they be diagnosed with an STI. The majority (97.5%) were not inclined to tell a partner face to face if they themselves were diagnosed with STIs, but they preferred personal delivery of the PN slip to the sexual partner. Lack of communication about sexual issues because of stigma and judgement has implications for STI treatment and control.

There was a vast difference between perceived use of referral slip from a sexual partner and the acceptability of PN by using SMS from the HCWs (93% vs. 62.7%). Evidence from studies indicates that the uptake of electronic PN has been slow, especially in settings where the impact may be greatest.^27^ In the current study, over a third of minibus taxi drivers were not in favour of PN by SMS from a HCW. They preferred telling partners face to face, as they cited that the SMS was not a reliable method and had a possibility of causing conflict in the relationship. These findings are in consensus with other studies.^27,28^ Whilst SMSs are likely to increase notification to partners who may not be notified otherwise, research shows that individuals are more inclined to seek services when they are notified face to face rather than anonymously.

Only a quarter of taxi drivers indicated personal preference for using SMS from healthcare providers, whereas 24% preferred patient-initiated PN referral slips and 51% preferred face-to-face notification. The use of SMS has the potential to reduce costs, expand coverage and increase efficiency of both provider and patient-initiated PN services.^12,27,29^ With some evidence of acceptability of provider-initiated PN by using SMS, and where more than three out of four people in the world have mobile phones,^12,27^ it would be beneficial as a complement to PN slips in South Africa. With the use of SMS, healthcare providers and patients can use mobile phones and text messaging services to contact partners in ways that were not possible before increased access with communication technologies.^27,29^ However, it is important for healthcare providers to take note of the different preferences for PN to provide patients with more options to choose a PN method that is best suited to their relationships and circumstances.^28,29^

## Limitations

The study findings should be interpreted after taking the following limitations into consideration. The study sample consisted of a sample of taxi drivers in a district of one province in South Africa, so the findings cannot be generalised to all minibus taxi drivers. This was a cross-sectional survey based on a scenario assessing the perceptions of usage rather than actual usage and acceptability of PN as in randomised controlled trials. We could not ask for the reasons for the high acceptability of SMS provider-initiated PN because this was a formative assessment of acceptability using a hypothetical scenario; therefore, these findings do not provide an explanation of why the study sample would tell the partner of the STI diagnosis. The other limitation is that the sample consisted of men only, thus the views of women on PN were not assessed.

## Conclusion

We found that a high proportion of the minibus taxi drivers understood the importance of notifying sexual partners once diagnosed with an STI. Their preferred choice of PN was the face-to-face approach; however, there were similar levels of preference of patent-initiated PN and acceptability of provider-initiated notification. This implies that both the patient- and provider-initiated PN are necessary for STI control and STI notification.

The findings provide healthcare providers with an opportunity to design PN of STI messages to educate partners about STIs and encourage them to access treatment. This also suggests that the PN protocol should be flexible to allow healthcare providers to provide patients with the option of using a PN method that is best suited for the patient relationship and circumstances.

It is therefore recommended that provider-initiated PN by using SMS should be considered an additional approach to the existing referral slip-based PN approach. An additional approach will provide options for patient to choose their preferred method of notifying sexual partners about potential STIs. Reproductive health programmes such as STI treatment and control should explore more counselling and encourage STI couple counselling and treatment sessions.
